# A Community to Care for All Generations: A University, Municipality, and Neighborhood Collaboration

**DOI:** 10.1093/geroni/igaf122.3239

**Published:** 2025-12-31

**Authors:** Yuka Sumikawa, Mana Shirouchi, Hiroshige Matsumoto, Kyoko Yoshioka-Maeda, Ayumi Igarashi, Noriko Yamamoto-Mitani

**Affiliations:** University of Tokyo, Bunkyo-City, Tokyo, Japan; The University of Tokyo, Tokyo, Tokyo, Japan; The University of Tokyo, Tokyo, Tokyo, Japan; The University of Tokyo, Tokyo, Tokyo, Japan; The University of Tokyo, Tokyo, Tokyo, Japan; The University of Tokyo, Bunkyo, Tokyo, Japan

## Abstract

Japan is facing unprecedented challenges to sustain the care delivery system, due to the sharp population decline and the increase of super-aged population. Sustainable care system cannot be actualized without working across research, policy planning, and community design with residents, government agencies, and universities in an interdisciplinary effort. Innovative new perspectives on care and well-being are needed. Our university is implementing a Community Based Participatory Research (CBPR) aimed at extending well-being and happiness expectancy of people in the community. At the heart of this project is a hub within our campus where we aim at various intergenerational community activities and exchange. We conducted a “Community Building Workshop” with community residents, public health representatives, and university members. This collaborative workshop brought together different stakeholders to share perspectives on community needs and explore ideal campus-community relationship. The workshop helped participants understand and identify meaningful roles university campuses could play in communities. Through lectures, campus tours, and World Café sessions, insights were gathered from 40 participants, half of whom were local residents. Attendees expressed various expectations for a community-accessible campus, including spaces for casual health consultations, care education opportunities, disaster prevention functions, and intergenerational interaction venues. Post-workshop survey results showed over 80% of respondents were satisfied with the experience. Through this collaborative workshop, we identified invaluable insights that will guide our efforts to create an effective intergenerational campus-community partnership model contributing to sustainable care systems in Japan with its changing demographic profile. This study was approved by our affiliated university ethics committee (2024314NI).

